# Patterns of Meniscal Injuries in Adults Aged 35 and Older: A Retrospective Analysis of Surgical Cases

**DOI:** 10.3390/medicina61040643

**Published:** 2025-04-01

**Authors:** Monica Şuşan, Andreea Maria Cristea, George Andrei Drăghici, Dragoş Vasile Nica, Sorin Florescu, Cosmin Grațian Damian

**Affiliations:** 1 Department of Internal Medicine, Centre for Preventive Medicine, Eftimie Murgu Square No. 2, 300041 Timișoara, Romania; susan.monica@umft.ro; 2 Research Center for Pharmaco-Toxicological Evaluations, Faculty of Pharmacy, “Victor Babes” University of Medicine and Pharmacy Timisoara, Eftimie Murgu Square No. 2, 300041 Timisoara, Romania; andreea.cristea@umft.ro (A.M.C.); nicadragos@gmail.com (D.V.N.); 3 Faculty of Pharmacy, “Victor Babeș” University of Medicine and Pharmacy Timisoara, Eftimie Murgu Square No. 2, 300041 Timisoara, Romania; 4 The National Institute of Research—Development for Machines and Installations Designed for Agriculture and Food Industry (INMA), Bulevardul Ion Ionescu de la Brad 6, 077190 București, Romania; 5 Department XV, Discipline of Orthopedics-Traumatology, “Victor Babeș” University of Medicine and Pharmacy Timișoara, Piața Eftimie Murgu 2, 300041 Timișoara, Romania; florescusorin@yahoo.com; 6 Faculty of Medicine, “Vasile Goldiș” Western University of Arad, Bulevardul Revoluției 94, 310025 Arad, Romania; damian.gratian@uvvg.ro

**Keywords:** meniscal injuries, knee joint pathology, meniscus rupture, surgical treatment

## Abstract

*Background and Objectives*: Knee joint injuries incur substantial healthcare and socioeconomic burdens worldwide. The connection between various demographic and clinical factors and meniscal injury patterns in patients undergoing surgery for meniscal rupture remains underexplored, especially in Eastern European cohorts. This study aimed to determine the influence of age, sex, and history of previous meniscal rupture on the patterns and types of knee joint injuries in adults aged 35 years and older undergoing surgery. *Materials and Methods:* A single-site exploratory retrospective analysis was conducted on 420 Romanian patients. The age of 35 years was selected as a cut-off for recruiting patients, as it marks the typical age at which early degenerative changes in the musculoskeletal system begin to emerge. Nonparametric/frequency analysis was applied to datasets stratified based on injury type—medial meniscal damage (MMD), lateral meniscal damage (LMD), and any patellar damage (APD). Logistic regression was used to determine influential predictors, including age, sex, and history of meniscal rupture. *Results*: Surgery was performed at a significantly younger age in patients with previous meniscus rupture (*p* < 0.001), but at a significantly older age in patients with co-occurring patellar lesions (*p* = 0.048). Men tended to be younger at the time of first surgery or any reoperations (*p* = 0.054) and displayed LMD significantly more often than MMD (*p* = 0.023). Significant differences existed in the distribution of different tear types in LMD (*p* < 0.001) and MMD (*p* < 0.001), with bucket handle tears and parrot beak tears being the most common. Male sex was associated with significantly higher odds of LMD (*p* = 0.046). Patients with previous meniscal rupture had a significantly and approximately threefold higher likelihood of presenting with MMD (*p* = 0.003). *Conclusions*: Age, sex, and history of meniscal rupture significantly influence the patterns and prevalence of knee injuries in adults aged 35 years and older. These findings reveal a dynamic interplay between demographic factors and knee joint pathologies, providing a foundation for targeted prevention and treatment strategies. Future studies should expand to larger, diverse populations to refine these insights.

## 1. Introduction

Knee joint injuries are a major contributor to movement impairment, such as gait disturbances, limited range of motion, and instability [1,2,3]. This prevalent and debilitating yet often overlooked condition typically affects the menisci (medial and lateral) and patella—structures pivotal to knee functionality and stability [4]. The medical costs, reduced productivity, and long-term care needs associated with this ailment pose a substantial burden on healthcare and economic systems worldwide [5,6]. As an example, in Italy, osteoarthritis-related knee arthroplasties alone cause up to 1 million lost workdays for patients younger than 65 [7]. Moreover, medical expenses related to knee injuries in Australia exceed AUD$3.5 billion per year [2].

Meniscal injuries have attracted considerable research interest, with much of the research focusing on sport-related damage and degenerative joint disease [2,3,8]. Patellar lesions—often manifesting as part of patellofemoral pain syndrome or osteoarthritis—are also common causes of knee discomfort and functional impairment [9,10]. Not only is aging a critical driver of knee joint pathologies [8,11], but the cause of damage also differs by age. Younger individuals are prone to sustaining acute, traumatic injuries, particularly during high-impact activities (e.g., impact sports, running, jumping) [12]. Older adults, by contrast, are predisposed to degenerative patterns of meniscal and patellar damage (e.g., cartilage thinning, osteophyte formation, joint space narrowing) [13].

Meniscal tears are a common type of meniscal injury, broadly referring to any structural damage to the meniscus and ranging from minor lesions to substantial damage [14]. A meniscal rupture can be viewed as a severe and advanced form of a meniscal tear, typically involving a complete or near-complete structural failure of this fibrocartilage [15]. Associated with pain, swelling, stiffness, and difficulty moving the knee, this frequent orthopedic pathology is primarily caused by lateral trauma (e.g., contact sports, motor vehicle collision) or internal factors (e.g., age, predisposition) [16]. Although patellar lesions can accompany meniscal tears in both traumatic knee injuries and degenerative changes [17,18,19], little information exists about their co-occurrence in a real-world clinical setting.

A substantial number of studies on patellar/meniscal damage has focused on specific age brackets rather than entire populations [1,2,10,17,20,21]. This targeted approach is often used because the varying biomechanical and physiological features differ across age groups, affecting the outcomes of their treatments. For example, age exerts a strong effect on meniscus cellularity, with patients aged 40 years and above having significantly fewer meniscal cells than younger patients [22]. As a result, younger and older cohorts of patients display distinct trends and outcomes in meniscal/patellar repair and treatment [23,24,25]. Clinical evidence also indicates that reoperation accounts for a notable percentage of cases undergoing surgical treatment of meniscus rupture, especially in cases with complex injuries or specific patient demographics. In this regard, a study in a military population found a reoperation rate of 13.6% following meniscal repair, with a median time to reoperation of 1.1 years. Factors such as younger age, military service branch, and rank were significant risk factors for reoperation [26]. Nonetheless, the connection between age, sex, and reoperation rate for meniscal rupture remains largely unexamined [27].

The specific pattern of damage in meniscal rupture involves distinct types and locations of tears, often associated with other types of knee joint injury [28]. However, the incidence of different tear patterns (e.g., radial, horizontal, bucket handle, parrot beak) in subjects with first-time versus previous meniscus rupture is understudied. Sex- and age-dependent variations in the occurrence, type, and severity of meniscal tears are also not comprehensively understood. Moreover, the very small amount of data available on knee joint injuries in Eastern Europe limits our understanding of intra-European differences in injury mechanisms, healthcare access, and treatment outcomes [28]. This is an important gap, as the causes of meniscus injuries may vary regionally, influenced by factors such as lifestyle, occupational demands, and sports participation. For example, American football and basketball are associated with an elevated incidence of medial meniscal tears in the USA [29]. As in North America, meniscal damage is prevalent among athletes in Europe. However, its incidence and management vary across different regions depending on the predominant sports played, such as football, rugby, skiing, or basketball, as well as differences in training intensity and healthcare systems [30]. The military environment also exhibits a higher rate of meniscal injuries, with specific demographics and activity levels accounting for this increased incidence [31]. Moreover, we note that studies focusing on adults aged 35 years and older are scarce, although these patients lie at the intersection of acute and degenerative pathologies, contribute substantially to healthcare burdens, and display distinct treatment needs and outcomes [23,24,25,26,27,28].

In this context, we aimed to determine the influence of key patient-related factors—specifically age, sex, and history of previous meniscal rupture—on the patterns and types of knee joint injuries observed in adults aged 35 years and older undergoing meniscal surgery. Understanding these associations in a robust sample size of over 400 patients contributes to a better understanding of associated risk profiles and lays a foundation to support more personalized approaches to diagnosis, treatment planning, and rehabilitation. Expanding this research line could also provide valuable insights into the epidemiology of knee joint injuries within Eastern European populations, where region-specific factors such as occupational demands, healthcare access, and activity levels may influence injury patterns.

## 2. Materials and Methods

### 2.1. Study Design

This single-site, exploratory study was a retrospective cross-sectional analysis of patients presenting for surgical treatment of meniscal rupture. Data were extracted from clinical records of subjects evaluated at Arad County Emergency Clinical Hospital (Romania) between 1 January 2016 and 1 February 2025. The study was approved by the Institutional Ethics Committee (approval number 92/20.01.2025) of the aforementioned hospital and adhered to the principles of the Declaration of Helsinki. Informed consent was obtained from all participants in accordance with ethical standards.

### 2.2. Patient Cohort

The study population included adults aged 35 years or older. The age of 35—applied here as a threshold for recruitment—is important for the incidence of orthopedic trauma, as several key biological, lifestyle, and environmental factors converge at this point in life. Thus, individuals in their mid-30s frequently maintain a vigorous exercise regimen and occupy physically demanding jobs, having a higher likelihood of traumatic knee injuries [23,24,25]. Bone resorption also begins to surpass bone formation during this time frame, serving as a major risk factor for reduced bone density and fractures [24]. In addition, sarcopenia—the gradual, age-related loss of muscle mass and strength—often begins within the same age bracket, affecting joint stability and increasing the risk of falls and injuries [26,27]. Importantly, healing processes gradually slow down with advancing age as well, resulting in prolonged recovery times from injuries and a higher likelihood of complications [28].

Meniscal rupture was defined as a meniscus tear with complete or near-complete structural failure of the meniscus. The present study enrolled patients who underwent surgical repair for meniscal injuries. These patients had either medial meniscal damage (MMD), lateral meniscal damage (LMD), or both types of meniscal damage simultaneously, with or without associated patellar lesions (APD). Patients with previous meniscal injuries were included, provided that the current injury involved the same knee or an anatomically distinct area. Another important inclusion criterion was the availability of complete and detailed demographic and clinical data (including age, gender, history of meniscal injury, diagnosis, type of meniscal injury). This study prioritized pre-surgical factors, leaving BMI and outcomes for future analyses to avoid confounding or data variability.

The exclusion criteria included patients with missing or incomplete demographic, clinical, or injury-related data; cases with prior knee surgeries unrelated to meniscal or patellar injuries (to avoid confounding effects on injury patterns); individuals with systemic inflammatory disorders (e.g., rheumatoid arthritis, lupus) known to affect joint health; patients with congenital or developmental knee abnormalities (e.g., patellar maltracking, genu varum/valgum) that could predispose them to injuries unrelated to age; cases with severe osteoarthritis or advanced joint degeneration not linked to meniscal or patellar injuries; patients with polytrauma or concurrent systemic injuries (e.g., fractures) affecting overall joint function; individuals with extreme BMI values (<18.5 or >40); patients with neuromuscular conditions (e.g., Parkinson’s disease, multiple sclerosis) that could affect gait or predispose them to knee injuries unrelated to meniscal damage; individuals with uncontrolled chronic diseases (e.g., diabetes, cardiovascular conditions) that might impact healing or outcomes; and patients with significant prior injuries or degenerative changes in the contralateral knee that could lead to compensatory biomechanical alterations. A total of 420 patients met the inclusion criteria and were included in the analysis. This is well above the cut-off value of 200–300 individuals recommended by the clinical literature for conducting a robust exploratory study [32].

### 2.3. Statistical Analysis

All statistical analyses were performed with Statistica software, version 8 (StatSoft Inc., Tulsa, OK, USA). A significance threshold of *p* < 0.05 was used for all tests [33]. Continuous variables (i.e., age) were summarized as medians with lower and upper quartiles. Categorical variables (e.g., gender, injury type) were presented as frequencies with the corresponding percentages. Intra-strata differences in median age across different types of knee joint injury (i.e., with vs. without damage) were determined using Mann–Whitney U tests [34]. The corresponding differences in sex distribution were assessed with Chi-square (χ^2^) tests based on 2 × 2 contingency tables. A similar approach was used for median age and sex distribution in patients stratified based on the history of meniscal rupture [35].

A χ^2^ test with 2 × 2 contingency tables was used to identify whether there were significant differences in the incidence of medial meniscal damage and lateral meniscal damage. The patients from each strata were then divided into subjects without meniscal damage and patients with meniscal damage, and the latter into three further groups—the most frequent type of damage, the second most common, and others. This aimed to differentiate between the distribution of different tear patterns in medial meniscal damage and lateral meniscal damage. Statistical analysis was conducted with χ^2^ tests based on a 4 × 2 contingency tables.

Logistic regression—a method appropriate for dichotomized datasets—was then applied on different types of knee injury (presence or absence of MMD, LMD, and APD as outcomes) to identify the effects of sex, age, and medical history (as factors). In the case of dependent variables (outcomes), their presence was coded with 1 and their lack with 0. Sex was binary-coded; the reference group was females (coded as 0), while males (coded as 1) were compared against this baseline. For HMR, a documented history of previous meniscal rupture was coded 1. Variable age was included as a continuous predictor, and hence was not categorized. The direction and strength of the association between significant predictor variables and outcomes were evaluated using odds ratios [36].

## 3. Results

Over the 10-year analysis period, 420 adults aged 35 years and above underwent surgical treatment of meniscal rupture. The median age of the entire cohort was 45 years (39; 53). Out of all patients, 264 and 156 were males (62.85%) and females (37.15%), respectively. Male patients were significantly younger than female patients at the time of receiving surgical treatment (Mann–Whitney test, 40 years (36; 51) vs. 47 years (41; 55), *p* < 0.001). Median ages with lower and upper quartiles and the number of individuals per each type of knee joint injury are presented in Table 1. No significant inter-strata differences in age were found for medial meniscal damage and lateral meniscal damage (Table 1). Individuals with patellar lesions, however, were significantly older than those without this condition (Table 1). Notably, 80 adults (19.05%) showed both medial meniscal damage and lateral meniscal damage.

Sex distribution depending on the type of knee joint injury is given in Table 2. The incidence of medial meniscus damage was not affected by sex (Table 2). In contrast, significant differences in sex distribution existed between patients with and without lateral meniscus lesions, the former group having a much higher proportion of males (Table 2). Gender distribution was, however, similar for any patellar damage (Table 2).

Median age was significantly lower across patients with previous meniscal rupture (Mann–Whitney test, 40 years (37; 50) vs. 47 years (40; 54), *p* < 0.001). The incidence of first-time meniscal rupture was higher in males (*n* = 182, 58.71%) than in females (*n* = 128, 41.29%). This scenario was also applicable to patients with previous meniscal rupture, with males *(n* = 82, 74.55%) outnumbering females threefold *(n* = 28, 25.45%). Inter-strata differences in sex distribution exhibited a noticeable trend toward significance (χ^2^ test, *p* = 0.054).

The incidence of different types of meniscal tear configuration are presented in Table 3. We identified significant differences in the distribution of different tear types of the lateral meniscus (χ^2^ test, *p* < 0.001) and medial meniscus (χ^2^ test, *p* < 0.001). From the entire cohort, a significantly larger proportion of patients had medial meniscus damage than lateral meniscus damage (χ^2^ test, *p* < 0.001). Bucket handle tears and parrot beak tears were the most frequent type of lesions in both cases, with the former being twice as common (Table 3). The incidence of these types of tears for medial meniscal damage was also at least twofold higher than that for lateral meniscal damage (Table 3). Other types of meniscal tears included double bucket-handle tears, transverse tears, horizontal tears, and bell clapper tears, either individually or in combination.

Table 4 summarizes logistic regression results for predicting three outcome variables (MMD, LMD, APD) based on the predictors: age, sex, and history of meniscal rupture. The table includes the *p*-values (significance) and the corresponding estimates (coefficients) for each predictor and outcome. Age did not show any significant association with any form of knee joint injury (Table 4). However, sex was a significant predictor for the incidence of lateral medial damage, with males being more likely than females to display this condition. Moreover, history of meniscal rupture was a significant predictor of medial meniscal damage (Table 4). Patients with previous meniscus rupture had a significant and approximately threefold increase in the likelihood of having this pathology (Table 4). In contrast, no significant associations were detected between sex, age, or history of meniscal rupture and the other outcome variables.

## 4. Discussions

A focus on specific age brackets is prevalent in investigations of knee-joint conditions [1,2,10,17,20,21]. However, few studies have focused on individuals aged 35 years and older. This transitional point is an important hallmark in clinical orthopedic literature, as it marks the onset of age-related changes in knee joint health and a rapid increase in the risk of knee lesions and the need for surgical interventions [37]. The present findings contribute to the advancement of knowledge of knee joint disorders by filling an important gap in data from Eastern Europe, exploring the impact of prior meniscus ruptures on re-injury risk, and investigating the potential co-occurrence of meniscal and patellar injuries during aging. We found that men underwent surgical treatment for meniscal rupture at a significantly younger age than women. Several causes may account for these findings. First, men tend to engage in high-impact sports and physically demanding activities at younger ages. This increases their risk of acute trauma-related meniscus tears, often necessitating earlier surgical intervention [38]. Second, estrogen is important for collagen metabolism and joint stability, potentially postponing structural deterioration in knee joints during reproductive years [39]. Third, gender differences in healthcare-seeking behavior may be linked to the observed age disparity. For example, men may seek immediate care following sports injuries. Women, by contrast, could postpone treatment for nontraumatic or less severe knee pain, thus requiring later surgical intervention [40]. This behavior is hazardous, because postponement of surgery beyond one year increases the risk of irreparable medial meniscal tears in women [41]. These factors offer a multi-faceted explanation for the earlier need for surgical treatment for meniscal rupture in male patients compared to female patients.

Individuals with meniscus rupture associated with patellar damage tended to be of significantly greater age. To our knowledge, such data have not yet been reported. There is nonetheless pertinent evidence for age-dependent degeneration in the patella–patellar tendon complex. Therefore, it was demonstrated that the internal milieu of the patellar tendon is altered with aging [42]. Age-related meniscal extrusion increases contact stress on the patella, contributing to concurrent damage [43]. The thickness of the patella and its associated tendons also decreases with age [44,45]. These insights reveal the interconnected nature of degenerative changes in the patella and meniscus regions.

The higher likelihood of lateral meniscal damage in males versus females is in line with literature data. For example, Kluczynski et al. found a prevalence of lateral meniscal tears nearly twice as high in men versus women (46% vs. 27.8%) in a prospective study with 689 patients [46]. Higher prevalence among men relative to women was reported by other authors as well, e.g., by Dandy et al. for a cohort of 1000 symptomatic meniscus injury patients [47], Wu et al. in a meta-analysis based on data from 6589 individuals [48], and Snoeker et al. in a meta-analysis with 7358 participants [49]. These findings may originate from structural and functional differences between the two menisci. The lateral meniscus allows for more flexibility, as it is more mobile and less constrained than the medial meniscus [50]. This mobility, however, comes at the price of higher susceptibility to injury during rotational movements, and high-speed and high-impact activities. Moreover, male patients are more likely to engage in contact sports (e.g., football, rugby) and physically demanding activities compared to female patients, as already mentioned above. The combination of anatomical features (i.e., larger tibial plateau, increased meniscal volume and surface area) and typically higher biomechanical demands also makes men more prone to lateral meniscal damage compared to women [51].

Our results provide a nuanced perspective on the interplay between age, sex, and reoperation rate for meniscus rupture [27]. Specifically, patients with prior meniscus rupture were significantly younger at the time of surgery compared to first-time patients. Indeed, young individuals exhibit an elevated risk of re-injury in the previously compromised meniscus [16], as they engage more often in active sports and/or work in physically demanding occupations [52]. On the other hand, altered biomechanics following an initial meniscal rupture or surgical intervention act as an additive factor increasing susceptibility to future damage [53]. The loss of meniscal tissue reduces the stability of the knee joint and its shock-absorbing capacity, causing greater wear and tear to the remaining meniscus. It is also noteworthy that traumatic mechanisms (e.g., twisting, pivoting) are common causes of meniscal ruptures in younger patients [2,13]. Typically acute, these injuries often require surgical intervention to restore normal function, further increasing the likelihood of subsequent ruptures.

Male individuals constituted the majority in both first-time patients and those with a recurrent meniscus rupture. This predominance of men over women most likely reflects a combination of anatomical, biomechanical, behavioral, and activity-related factors rather than the effect of a single factor. More precisely, higher engagement in high-risk activities, biomechanical stressors, hormonal and degenerative differences, and a tendency to return to demanding activities post-injury contribute meaningfully to the observed trend [13,16,38,39,49,54].

We identified a significantly higher prevalence of medial meniscal damage versus lateral meniscal damage in patients aged 35 years and older undergoing surgical treatment for meniscus rupture. Published evidence supports our findings. For example, Baker et al. reported that the incidence of medial versus lateral meniscus injury was 81 vs 19% in a cohort of 1515 patients, with a sex ratio of three males to one female [29]. Jackson et al. observed a similar trend when analyzing the patterns of meniscal damage in a pediatric population over a 16-year period [55]. Importantly, literature data lend support for a progressive, age-dependent increase in the prevalence of medial meniscus tears among individuals over 30 years old [56]. These injuries are often degenerative, being associated with conditions like obesity and varus deformity—which are also frequently observed in this demographic [57]. Younger populations, particularly those under 20 years and in acute settings, typically display isolated lateral meniscal injuries [58,59].

Concordant with previous findings, the bucket handle tears and the parrot beak tears were the most common types of meniscal tears [60,61,62]. These injuries occurred more frequently in the medial meniscus than in the lateral meniscus. The medial meniscus, being less mobile and more restricted due to its stronger attachment to the tibial plateau and joint capsule [44], is less capable of absorbing substantial compressive, rotational, or torsional forces. Consequently, it is more prone to developing bucket handle tears and parrot beak tears [60,62]. On the other hand, the medial meniscus is more frequently targeted in diagnostic evaluations due to its association with symptoms like joint locking and instability [53], thereby leading to an apparent overrepresentation of these tear types. Typically impacting athletes who twist their knees during sports activities, the bucket handle tears present more pronounced symptoms and achieve better outcomes after surgery versus other types of tears [60]. Partial meniscectomy is generally preferred over total meniscectomy to help maintain knee joint stability and function, reduce the risk of osteoarthritis, and improve long-term outcomes. The same approach is also recommended in the case of parrot beak tears [63].

A common practice in exploratory studies, the integration of nonparametric/frequency analysis with logistic regression enables scientists to refine the list of potential variables, ensuring statistically validated and clinically meaningful findings for future studies [64]. Here, logistic regression identified sex as a significant predictor, with males being more likely to experience lateral meniscal injuries. This is consistent with the results of frequency analysis. According to the same logistic model, a history of meniscus rupture was another influential factor, increasing the odds of medial meniscal damage, but not lateral meniscal damage, in individuals with prior meniscus injuries. The observed association can be attributed to a combination of anatomical factors (e.g., reduced mobility of medial meniscus), biomechanical drivers (e.g., greater exposure to altered biomechanics post-injury), and activity-related determinants (e.g., higher stress from physical activities in younger individuals), as already described above [13,16,38,39,48,49]. On the other hand, logistic regression failed to identify any significant predictors for patellar damage, aligning partially with the nonparametric findings (lack of sex or history association) but not confirming the age-related trend. This discrepancy suggests that additional factors influencing patellar damage (e.g., inflammatory conditions, trauma, degenerative changes) may not have been captured in the current model.

Overall, our results provide valuable insights into the patterns of meniscus injuries among individuals aged 35 years and older. Surgery is typically performed at a younger age in patients with previous meniscus rupture, but at an older age in patients with co-occurring patellar lesions. Men tend to undergo surgical intervention and their first reoperation at a younger age. Male patients are also more prone to displaying lateral meniscal damage. Furthermore, previous meniscus rupture is associated with a higher likelihood of medial meniscus damage. Bucket handle tears and parrot beak tears are among the most common types of meniscus lesions, occurring more frequently at the medial meniscus than at the lateral meniscus.

The present study is not without limitations. As a retrospective study conducted at a single institution, the present investigation is prone to selection bias and limited generalizability of the findings to other populations or healthcare settings. This design, however, allows for the analysis of extensive datasets over time, essential for identifying trends and patterns. This single-site approach also ensures uniformity in surgical and diagnostic practices, reducing data variability [65]. Another important drawback is the exclusive reliance on clinical records, as these documents might not include all relevant variables (e.g., detailed activity levels, comorbidities). Although clinical records may indeed lack certain variables, they provide a standardized and reliable source of information on diagnosed conditions, treatments, and outcomes. This ensures consistency across the entire dataset, thus minimizing the risk of self-reporting biases often associated with activity levels or comorbidity data [66]. As a major limitation, we also note the lack of data on long-term outcomes of the injuries or surgeries, restricting the possibility to determine the progression of degenerative changes or the success of treatments. While the exploratory nature of the present study is a solid base for future longitudinal research, its inherent limitations—such as the lack of temporal data and causative inferences—cannot be overlooked. However, this preliminary analysis is crucial in identifying key variables and trends that warrant further investigation [67].

Future longitudinal studies can build upon these findings to assess long-term outcomes, including recovery rates, functional status, and reoperation rates, while also exploring causal relationships and refining clinical interventions. In addition, multi-center studies with diverse populations are needed to validate these findings and explore the impact of lifestyle factors, comorbidities, and diagnostic practices on the prevalence and treatment outcomes of meniscal injuries. Moreover, certain aspects remain unclear, such as the precise mechanisms underlying the progression of concurrent meniscal and patellar injuries with aging and the role of less-studied factors, like genetic predisposition or chronic low-grade inflammation, in influencing these injury patterns. Further research is therefore needed to address these gaps and develop comprehensive prevention and treatment strategies.

## 5. Conclusions

Patients aged 35 years and older with a previous meniscus rupture were significantly younger at the time of surgery than those without a history of meniscal rupture. The opposite trend was observed for patients with co-occurring patellar lesions. The prevalence of medial meniscal damage was significantly above that of lateral meniscal damage. Men tended to be younger than women at the time of surgery and revealed a significantly higher risk for lateral meniscal injuries. Patients with a history of prior meniscal rupture had a significantly increased—approximately threefold—likelihood of exhibiting medial meniscal damage. Bucket handle and parrot beak tears were the most frequently observed meniscal injuries, predominantly affecting the medial meniscus. These results support an intricate relationship between degenerative and traumatic factors in knee joint injuries.

## Figures and Tables

**Table 1:** Median age by type of knee joint injury.

Knee Joint Damage	With Damage		Without Damage		*p*
	Median Age	*n*	Median Age	*n*	
Medial meniscus damage	46 (39; 53)	202	44 (38; 53)	218	0.878
Lateral meniscus damage	42 (38; 49)	76	46 (39; 54)	344	0.180
Any patella damage	46 (39; 53)	366	39 (36; 51)	54	0.048 *

Data are given as median values with lower and upper quartiles (in parentheses). Marked values (*) indicate significant differences (Mann–Whitney test, ***—*p* < 0.001, **—*p* < 0.01, *—*p* < 0.05).

**Table 2:** Distribution of patients by sex and type of knee joint injury.

Knee Joint Damage	With Damage		Without Damage		*p*
	Male	Female	Male	Female	
Medial meniscus damage	126 (56.75%)	76 (33.25%)	138 (63.30%)	80 (36.70%)	0.889
Lateral meniscus damage	60 (78.91%)	16 (21.06%)	204 (59.30%)	140 (41.70%)	0.023 *
Any patella damage	42 (77.77%)	12 (32.23%)	222 (60.65%)	144 (29.35%)	0.085

Data are given as frequencies with the corresponding percentages (in parentheses). Marked values (*) indicate significant differences (χ^2^ test, ***—*p* < 0.001, **—*p* < 0.01, *—*p* < 0.05).

**Table 3:** Distribution of different tear types in medial meniscus and lateral meniscus.

	With Damage			Without Damage
	BH	PB	Other	
Medial meniscus	78 (18.57%)	38 (9.05%)	74 (17.61%)	230 (54.77%)
Lateral meniscus	26 (6.19%)	8 (1.91%)	20 (9.52%)	173 (82.38%)

BH, bucket handle tear; PB, parrot beak tear. Data are given as frequencies with the corresponding percentages (in parentheses).

**Table 4:** Logistic regression analysis: predictors for knee joint injuries.

	OR (95% CI)	*p*	β	SE	Wald (Z)
	Medial meniscal damage				
Age	1.14 (0.63; 2.09)	0.621	0.131	0.303	0.43
Sex	0.99 (0.96; 1.02)	0.557	−0.010	0.015	−0.65
HMR	2.86 (1.45; 1.56)	**0.003** ***	1.050	0.338	3.11
	Lateral meniscal damage				
Age	0.43 (0.13; 1.03)	0.335	−0.847	0.451	−1.88
Sex	1.09 (1.01; 1.16)	**0.046** *	0.086	0.037	2.32
HMR	0.87 (0.39; 1.93)	0.695	−0.140	0.396	−0.35
	Any patellar damage				
Age	1.92 (0.71; 5.14)	0.153	0.653	0.445	1.47
Sex	0.96 (0.91; 1.01)	0.179	−0.041	0.026	−1.55
HMR	0.97 (0.39; 2.43)	0.794	−0.030	0.437	−0.07

HMR, history of meniscal rupture; OR (95% CI), odds ratio with 95% confidence interval; β, coefficient beta; SE, standard error; Wald (Z), Z-value from the Wald test. Odds ratios are presented as absolute values with lower and upper bounds of the 95% confidence interval (in parentheses). Marked bold values (*) indicate significant outcomes (logistic regression, ***—*p* < 0.001, **—*p* < 0.01, *—*p* < 0.05).

## Data Availability

All the data generated or analyzed during this study are included in this published article.
